# Trauma and posttraumatic stress disorder modulate polygenic predictors of hippocampal and amygdala volume

**DOI:** 10.1038/s41398-021-01707-x

**Published:** 2021-12-16

**Authors:** Yuanchao Zheng, Melanie E. Garrett, Delin Sun, Emily K. Clarke-Rubright, Courtney C. Haswell, Adam X. Maihofer, Jeremy A. Elman, Carol E. Franz, Michael J. Lyons, William S. Kremen, Matthew Peverill, Kelly Sambrook, Katie A. McLaughlin, Nicholas D. Davenport, Seth Disner, Scott R. Sponheim, Elpiniki Andrew, Mayuresh Korgaonkar, Richard Bryant, Tim Varkevisser, Elbert Geuze, Jonathan Coleman, Jean C. Beckham, Nathan A. Kimbrel, Danielle Sullivan, Mark Miller, Jasmeet Hayes, Mieke Verfaellie, Erika Wolf, David Salat, Jeffrey M. Spielberg, William Milberg, Regina McGlinchey, Emily L. Dennis, Paul M. Thompson, Sarah Medland, Neda Jahanshad, Caroline M. Nievergelt, Allison E. Ashley-Koch, Mark W. Logue, Rajendra A. Morey

**Affiliations:** 1 National Center for PTSD, VA Boston Healthcare System, Boston, MA USA; 2 Department of Biostatistics, Boston University School of Public Health, Boston, MA USA; 3 Department of Medicine, Duke Molecular Physiology Institute, Duke University Medical Center, Durham, NC USA; 4 VISN 6 MIRECC, Durham VA Health Care System, Durham, NC USA; 5 Brain Imaging and Analysis Center, Duke University, Durham, NC USA; 6 Department of Psychiatry, School of Medicine, University of California, San Diego, La Jolla, CA USA; 7 Center for Behavior Genetics of Aging, University of California, San Diego, La Jolla, CA USA; 8 Department of Psychological and Brain Sciences, Boston University, Boston, MA USA; 9 Center of Excellence for Stress and Mental Health, Veterans Affairs San Diego Healthcare System, La Jolla, CA USA; 10 Department of Psychology, University of Washington, Seattle, WA USA; 11 Department of Psychology, Harvard University, Boston, MA USA; 12 Minneapolis VA Health Care System, Minneapolis, MN USA; 13 Department of Psychiatry and Behavioral Sciences, University of Minnesota, Minneapolis, MN USA; 14 University of Sydney, Westmead, NSW Australia; 15 Brain Dynamics Centre, Westmead Institute of Medical Research, University of Sydney, Westmead, NSW Australia; 16 School of Psychology, University of New South Wales, Sydney, NSW Australia; 17 Brain Center Rudolf Magnus, Department of Psychiatry, University Medical Center Utrecht, Utrecht, The Netherlands; 18 Brain Research and Innovation Centre, Ministry of Defence, Utrecht, The Netherlands; 19 King’s College London, Social, Genetic and Developmental Psychiatry Centre, Institute of Psychiatry, Psychology and Neuroscience, London, UK; 20 King’s College London, NIHR Maudsley BRC, London, UK; 21 Department of Psychiatry and Behavioral Sciences, Duke University School of Medicine, Durham, NC USA; 22 VA Boston Healthcare System, Jamaica Plain, MA USA; 23 Department of Radiology, Martinos Center for Biomedical Imaging, Massachusetts General Hospital, Harvard Medical School, Charlestown, MA USA; 24 Department of Psychological and Brain Sciences, University of Delaware, Newark, DE USA; 25 Translational Research Center for TBI and Stress Disorders, VA Boston Healthcare System, Boston, MA USA; 26 Geriatric Research, Educational and Clinical Center, VA Boston Healthcare System, Boston, MA USA; 27 Department of Psychiatry, Harvard Medical School, Boston, MA USA; 28 Department of Neurology, University of Utah, Salt Lake City, UT USA; 29 Imaging Genetics Center, Stevens Neuroimaging & Informatics Institute, Keck School of Medicine, University of Southern California, Los Angeles, CA USA; 30 Queensland Institute for Medical Research, Berghofer Medical Research Institute, Brisbane, QLD Australia; 31 Departments of Psychiatry and Biomedical Genetics, Boston University School of Medicine, Boston, MA USA

**Keywords:** Psychiatric disorders, Clinical genetics, Predictive markers

## Abstract

The volume of subcortical structures represents a reliable, quantitative, and objective phenotype that captures genetic effects, environmental effects such as trauma, and disease effects such as posttraumatic stress disorder (PTSD). Trauma and PTSD represent potent exposures that may interact with genetic markers to influence brain structure and function. Genetic variants, associated with subcortical volumes in two large normative discovery samples, were used to compute polygenic scores (PGS) for the volume of seven subcortical structures. These were applied to a target sample enriched for childhood trauma and PTSD. Subcortical volume PGS from the discovery sample were strongly associated in our trauma/PTSD enriched sample (*n* = 7580) with respective subcortical volumes of the hippocampus (*p* = 1.10 × 10^−20^), thalamus (*p* = 7.46 × 10^−10^), caudate (*p* = 1.97 × 10^−18^), putamen (*p* = 1.7 × 10^−12^), and nucleus accumbens (*p* = 1.99 × 10^−7^). We found a significant association between the hippocampal volume PGS and hippocampal volume in control subjects from our sample, but was absent in individuals with PTSD (GxE; (beta = −0.10, *p* = 0.027)). This significant GxE (PGS × PTSD) relationship persisted (*p* < 1 × 10^−19^) in four out of five threshold peaks (0.024, 0.133, 0.487, 0.730, and 0.889) used to calculate hippocampal volume PGSs. We detected similar GxE (G × ChildTrauma) relationships in the amygdala for exposure to childhood trauma (rs4702973; *p* = 2.16 × 10^−7^) or PTSD (rs10861272; *p* = 1.78 × 10^−6^) in the *CHST11* gene. The hippocampus and amygdala are pivotal brain structures in mediating PTSD symptomatology. Trauma exposure and PTSD modulate the effect of polygenic markers on hippocampal volume (GxE) and the amygdala volume PGS is associated with PTSD risk, which supports the role of amygdala volume as a risk factor for PTSD.

## Introduction

The effects of psychological trauma and PTSD on society and individual functioning are immense as measured by a 150% increase in rates of unemployment, and a 60% increase in marital dysfunction, along with elevated rates of suicide, anxiety, and depression [1]. Prominent neurocognitive deficits that accompany PTSD include impaired memory, particularly fragmented autobiographical and trauma-related memories [2]. The hippocampus is involved in discrete aspects of memory encoding and consolidation. The deficits in episodic memory, contextual memory, and extinction failure suggest that the hippocampus plays a role in developing PTSD [3]. There is evidence of PTSD-associated structural differences in multiple subcortical regions, and a large multi-cohort consortium coordinated analysis of subcortical regions with a standard processing pipeline in 1868 subjects indicated that evidence was strongest for lower hippocampal volume in PTSD [4, 5].

It is unclear whether lower hippocampal volume in PTSD is a consequence of developing PTSD or the result of genetic or biological vulnerabilities. In monozygotic twins discordant for trauma exposure and PTSD, the trauma-unexposed co-twin had smaller hippocampal volume relative to PTSD-free twin pairs, suggesting that similarity due to shared genetics and/or early environment poses an increased risk for developing stress-related psychopathology [6].

There are multiple studies indicating that trauma itself is associated with reduced hippocampal volume [7], although other studies have not confirmed this [8–10]. A reduction in hippocampal volume due to trauma exposure may be due to the sensitivity of the hippocampus to stress hormones, and glucocorticoids in particular [11–13]. Our consortium recently reported that childhood trauma exposure has a negative association with hippocampal volume [4]. While this association was not significant after adjusting for PTSD, high collinearity between PTSD and trauma exposure presents a challenge. Genetics can also yield valuable information about causality, given that gene variants necessarily predate trauma exposure and psychopathology.

Aside from the hippocampus, the amygdala is the other subcortical structure most frequently implicated in PTSD symptoms, particularly intrusive symptoms [5]. The amygdala plays a central role in fear response and encoding of fear memories [14]. In our consortium study, lower amygdala volume was nominally associated with PTSD and childhood trauma, with a lower effect size than hippocampus and a significance level that did not survive multiple-testing correction [4, 5]. Other subcortical structures that play a role in PTSD neurobiology include the caudate [15, 16], nucleus accumbens, and thalamus [15, 16]. Microstructural alterations of the caudate, which are associated with PTSD, are hypothesized to disrupt striatal-dependent learning of fear associations [17]. Reduced caudate volume is associated with early life stress, while increased caudate and thalamic activity are associated with overgeneralization of conditioned fear in PTSD [15, 18].

Genetics clearly plays a role in the hippocampal structure. Twin studies indicate high heritability of hippocampal (h^2^ = 0.88) [19] and other subcortical volumes (*h*^2^ > 0.66) [20, 21]. Single variant genetic predictors of hippocampal volume are well established in non-clinical populations with genome-wide association studies (GWAS) [22–24]. Subcortical volumes and other quantitative traits derived from structural MRI are highly polygenic, meaning many genetic variants contribute small effects. Fortunately, polygenic scores (PGS) provide a simple, yet elegant, method to calculate an index of genetic risk for a target sample provided a well-powered GWAS of a related phenotype has been conducted in an independent discovery sample [25]. The PGS provides a single score that captures the effects of multiple genetic loci each weighted by the magnitude of its individual contribution to the phenotype. That is, PGSs are typically computed as the weighted sum of the effect size for each SNP multiplied by the additively coded number of alleles for that SNP (0–2). For polygenic phenotypes, PGS (for quantitative traits) and PRS (polygenic risk scores; for disease traits) explain a larger proportion of variance than any single risk variant. The volume of subcortical structures represents a reliable, quantitative, and objective phenotype to examine the role of genetic and environmental effects such as trauma and PTSD. Psychological trauma and PTSD represent potent environmental exposures that may interact with genetic markers, or act directly, to influence brain structure and function. Modulation of the PGS by environmental or disease contributors on the phenotype of interest can be tested in a straightforward manner [25]. While we may assume that PGS derived from a normative sample captures the most relevant genetic markers that influence subcortical volume, it is possible that heretofore unknown genetic markers interact with trauma and illness to impact subcortical volume.

Our goal was to use genetic variants previously associated with subcortical volumes in a normative discovery sample to compute PGS for hippocampus, amygdala, and other subcortical volumes in a target sample enriched for childhood trauma and PTSD. We accessed GWAS results on subcortical volume phenotypes from two GWAS studies conducted by the international consortium Enhancing NeuroImaging Genetics with Meta-Analysis (ENIGMA) [23, 26]. We calculated PGS in our target sample from the PTSD working group of the Psychiatric Genetics Consortium (PGC) [27] and ENIGMA-PTSD [28, 29]. We hypothesized that the environment, specifically trauma experienced during childhood, would modulate genetic factors as measured by PGS, to predict the volume of the hippocampus and amygdala. We hypothesized similar modulations of PGS by PTSD diagnosis. We investigated gene-environment interactions by (1) modeling PGS × E interaction using the PGS calculated from a large normative sample without PTSD and without trauma exposure (2) and modeling a G × E GWAS in our sample with PTSD cases and trauma-exposed controls. These complementary approaches were used to test hypothesized gene-environment interactions. Unlike childhood trauma, consistent information about adult trauma exposure was not available across cohorts. Given the overwhelming evidence from the published trauma and PTSD literature, we emphasized amygdala and hippocampal regions in our hypotheses over the other subcortical regions.

## Methods

### Cohorts

We meta-analyzed data originating from eight cohorts totaling 7580 subjects (4256 males and 3324 females) grouped in PTSD cases (670) and controls (6910). The present sample was comprised of data from Translational Research Center for TBI and Stress Disorders (TRAC) of US military veterans (Boston MA) [30], the Vietnam Era Twin Study of Aging (VETS) in US military veterans [31], VA Mid-Atlantic Mental Illness Research Education and Clinical Center (MIRE)/Duke University of post-9/11 era US military veterans (Durham NC) [32], United Kingdom BioBank (UKBB) of UK research participants [33], and PGC-PTSD [27]. The Psychiatric Genetics Consortium (PGC) PTSD sample itself consists of several cohorts including those from the University Medical Center Utrecht (Netherlands), Minneapolis VA Medical Center (USA), University of New South Wales (Australia), and University of Washington (USA). All subjects from these sites with complete data were included in our analyses.

The predictive ability of PGS can be greatly reduced when applied to people of different ancestry than that of the discovery sample. Thus, we included only genetically determined non-Hispanic white (NHW) subjects in our analyses. All PGC-PTSD sites were mega-analyzed, followed by meta-analysis with the remaining sites. This approach was adopted for several reasons. First, PGC-PTSD sites were genotyped on the same platform, which was the Illumina Psych Chip. In addition, the PGC-PTSD data set included sites with relatively small sample sizes that were most amenable to mega-analysis. Most importantly, all non-PGC-PTSD sites involved military or veteran subjects whose item-level data could not be directly shared and mega-analyzed due to relatively stringent privacy rules. Finally, studies such as VETSA and UKBB were substantially different in design, which discouraged mega-analysis. See Table 1 for descriptive statistics for each cohort.

**Table 1:** Descriptive statistics for included cohorts.

Cohort (site)	*N*	*N* male (%)	Mean age (SD)	*N* PTSD cases (%)^a^	*N* childhood trauma (%)^a^
PGC-PTSD	377	296 (78.5)	35.32 (11.34)	173 (45.9)	101 (50.8)
BETR	71	70 (98.6)	36.68 (10.00)	44 (62.0)	25 (36.8)
DEFE	174	165 (94.8)	32.86 (8.08)	64 (36.8)	N/A
BRY2	116	51 (44.0)	41.16 (12.34)	62 (53.4)	73 (63.5)
KMCT	16	10 (62.5)	13.87 (2.13)	3 (18.8)	3 (18.8)
MIRE	143	128 (89.5)	37.59 (9.86)	38 (26.6)	64 (44.8)
TRAC	169	158 (93.5)	31.18 (8.15)	92 (56.1)	38 (22.6)
UKBB	6570	3057 (46.5)	55.12 (7.42)	160 (3.2)	1271 (25.8)
VETS	321	321 (100.0)	62.01 (2.57)	34 (10.6)	196 (61.1)
Total	7580				

*DEFE* Defining Essential Feature of Neural Damage - VA Minneapolis HealthCare System, Minneapolis MN USA, *Meta* meta-analysis, *PGC-PTSD* Psychiatric Genetics Consortium-Posttraumatic Stress Disorder, *TRAC* Translational Research Center for TBI and Stress, Boston VA HealthCare System, Boston MA USA, *VETS* Vietnam Era Twin Study of Aging, San Diego VA Healthcare System, San Diego CA USA, *MIRE* Duke University and VA Mid-Atlantic Mental Illness Research Education and Clinical Center the Study of Post-Deployment Mental Health Study, Durham NC USA, *UKBB* United Kingdom BioBank, *BRY2* Bryant2 Sydney Neuroimaging, University of New South Wales Australia, *KMCT* Katie McLaughlin Child Trauma, Child Trauma and Neural Systems Underlying Emotion Regulation, University of Washington, Seattle WA USA, *BETR* Biological Effects of Traumatic Experiences, University Medical Center, Utrecht Netherlands.

^a^Percentages are computed based on the number of subjects with non-missing values.

Inclusion and exclusion criteria for each site are provided in the Supplementary Methods section of Nievergelt et al. [27]. Harmonized scales of childhood trauma were obtained from most sites (Supplementary Methods). The instrument used for PTSD diagnosis, criteria for PTSD diagnosis (DSM-IV or DSM-5), ascertainment of PTSD symptoms, childhood trauma, ancestry, scanner manufacturer/model, array chip for genotyping, imputation method, and imputation panel, differed by cohort as summarized in Supplementary Table 1 and detailed in Nievergelt et al. [27]. All subjects provided informed consent for procedures approved by local IRB and ethics committees.

### Imaging and segmentation

Scanner acquisition protocols and parameters for T1-weighted brain MRI scans for each cohort are provided in Supplementary Table 2. Quality control (QC) and segmentation of subcortical structures from structural MRI scans were performed using FreeSurfer [34] in conjunction with standardized ENIGMA protocols [4]. Volumes of left and right hemispheric subcortical structures were averaged for the nucleus accumbens, amygdala, caudate, hippocampus, pallidum, putamen, and thalamus. Phenotype associations with PGS were calculated for each subcortical structure, as detailed below.

### PGS calculation

The PGSs were calculated from genome-wide genotype data in each cohort using hard-call genotypes generated from imputed genotype data with an 80% certainty threshold. All SNPs with a missing rate greater than 5% or minor allele frequencies less than 5% were excluded from the PGS calculation. The PGS for each subcortical volume phenotype was calculated with PRSice [35] with default parameters for clumping. The clumping process was applied to eliminate redundant SNPs in high linkage disequilibrium (LD) with selected SNPs [36]. PGSs are generally computed from all SNPs with p-values from the discovery GWAS that fall below a selected threshold. As the maximizing threshold is generally not known in advance, we calculated PGSs for 1,001 thresholds ranging from *p* = 0.0001 to 1, in increments of 0.001, and used the most significant threshold in subsequent calculations. To avoid confusion, we refer to these as thresholds, rather than *p*-value thresholds when specifying the cutoff used for PGS calculation. The PGS for amygdala and hippocampal volume was computed from the discovery GWAS [22] along with the five remaining subcortical volumes. We additionally computed PGSs across the threshold grid based on the published hippocampal volume GWAS [26]. However, this meta-analysis method produced a *Z*-score rather than an effect size estimate, i.e. a *Z*-score weighted PGS. SNPs with GWAS results based on less than 80% of the maximum GWAS sample size (i.e., results based on a subset of the GWAS samples) were excluded from the PGS calculation. We examined the PGS computed from two published discovery GWAS. The first study by Hibar et al. [22] was conducted by the ENIGMA Consortium in *n* = 30,717 NHW subjects conducted separate GWAS for nucleus accumbens, amygdala, hippocampus, caudate, pallidum, putamen, and thalamus—hereafter referred to as the ENIGMA-GWAS-2015. The second study by Hibar et al. [26] was conducted jointly by ENIGMA and CHARGE Consortia in *n* = 33,536 NHW subjects that reported GWAS results for only hippocampus and hippocampal subfields—hereafter referred to as ENIGMA-GWAS-2017. Subjects in the current study were not part of either GWAS. We elected not to use the more recent subcortical volume GWAS by Satizabal et al. [37] because it included samples from UKBB that overlap with our target sample, which may cause spurious associations.

### Statistical analysis

Analyses were performed in R [38]. To aid in the interpretation of the effect size estimates, continuous predictors such as intracranial volume (ICV) and PGS, and the subcortical volumes tested as the response were standardized (mean = 0, SD = 1). Analyses were conducted separately by cohort. Association evidence for parameters of interest was aggregated across cohorts using a random-effects meta-analysis as implemented in R’s metafor package [39]. Forest plots were generated using the R package forestplot [40]. The analyses were carried out in the following three stages.

Stage 1: Maximizing PGSs: First, we fit linear models of the PGSs for each threshold in predicting the mean of the left and right hippocampal volume. Hippocampal volume was the dependent variable and PGS was the predictor variable along with covariates for ICV, sex, age, age [2], and principal components (PC), which characterize variance in genetic data that is derived from population substructure within our cohorts. The primary source of population substructure is diverse ancestry. For all cohorts except the VETS cohort, the standard linear model package in R (lm) was used to model hippocampal volume. As VETS was a twin study, we employed linear mixed models to assess subcortical volume to account for non-independence of twin pairs with the *lme4* package. The sex covariate was omitted in VETS, which is composed of only men. For the PGC-PTSD cohort, a covariate to adjust for the separate studies making up the cohort was included. For UKBB, 10 PCs were included in the model to control for the population substructure that was observed in scree plots. For the remainder of the cohorts, 4 PCs were included in the model. First, we examined the PGS computed from ENIGMA-GWAS-2015 at each of the 1001 thresholds to test associations with hippocampal volume in each cohort using a linear model with the covariates as described above. The summary results for the array of PGSs were then collated in a random-effects meta-analysis. A similar procedure was used to aggregate associations at each threshold for the PGS computed from ENIGMA-GWAS-2017. The hippocampal PGS most significantly associated with hippocampal volume across studies and thresholds was used in subsequent analyses. We used the maximizing PGS over the same 1001 thresholds based on the ENIGMA-GWAS-2015 for amygdala volume as our amygdala PGS for interaction testing purposes. The same procedure was used to select the most significant threshold for each of the five remaining subcortical volumes in subsequent analyses.

Stage 2: Subcortical volume PGS impact on PTSD: We ran analyses to test associations between the most predictive hippocampus and amygdala volume PGS with PTSD diagnosis. Each cohort was analyzed separately. For all of the cohorts except VETS, a generalized linear model was fit using R’s *glm* package with PTSD diagnosis (dichotomous) as the dependent variable, the PGS as predictor, along with age and sex as covariates. Generalized estimating equations (GEE) were used in the VETS cohort to fit models of PTSD allowing for correlated errors within twin pairs after mixed models failed to converge on subcortical volumes [41]. The *gee* package in R was used to fit these models with PTSD as the dependent variable, PGS as the predictor variable, and age as a covariate [42]. Estimates of the PGS effect on PTSD were aggregated across cohorts in a meta-analysis based on effect size estimates. After the hippocampus and amygdala volume, PGSs were tested for associations with PTSD, the other subcortical volumes were examined. See Supplementary Methods and Supplemental Results for a description of analyses of PTSD severity.

Stage 3: GxE PGS analysis: We tested whether the impact of genetic influences on the hippocampus and amygdala volumes were modulated by PTSD or childhood trauma. These models mirrored the linear models and covariates used in Stage 1. First, we assessed the interaction between the PGS and PTSD on hippocampal volume. PTSD and the interaction between PTSD and PGS were added to the model as described in Stage 1. The strengths of association for the interaction terms were combined across cohorts using meta-analysis. Next, we fit a similar interaction model for both hippocampus and amygdala volume by incorporating the main effect of childhood trauma and the interaction of PGS and childhood trauma. The main effects of potential confounders as covariates do not protect against spurious interactions due to these confounders. However, we did not include all possible G × covariate and E × covariate interaction terms in all models as suggested by Keller [43], as this may lead to model overfitting and loss of power. The likelihood of spurious interactions due to confounding effects with sex and population substructure is assessed with follow-up analyses and examining the pattern of association across cohorts.

In follow-up analyses, we also examined the impact of threshold on the observed interaction significance. Finally, we examined the other subcortical volume PGSs for interactions with PTSD and childhood trauma using the same procedure as used for the hippocampus and amygdala PGSs. See the Supplementary Methods and Results for a description of analyses of PTSD severity.

Stage 4: GxE GWAS: In addition, we performed a GxE GWAS to identify individual SNPs that interact with PTSD and childhood trauma to impact the hippocampus and amygdala volumes. PLINK analysis of the data was performed using linear models of subcortical volume [44]. The linear models in PLINK and the meta-analysis technique used here correspond closely to those used in the PGS models. One exception was the analysis of the VETS data, as PLINK does not account for twin correlation. For GxE GWAS purposes, one of each twin pair was dropped from the analysis, prioritizing the inclusion of twins with PTSD when possible, or randomly picking one of the predictors. The PTSD and childhood trauma variables were each examined respectively as predictors with covariates in the GxE GWAS corresponding to those described above for PGS models. Only SNPs with MAF > 5% in each cohort were analyzed. SNPs missing in more than two cohorts were excluded from the meta-analysis. Top associations from independent SNPs were selected using FUMA [45]. In addition to examining GxE effects at the genome-wide level, we also performed a candidate SNP investigation focusing on variants that have been associated with either the corresponding subcortical volume [37, 46] or PTSD, or with PTSD symptoms [47]. Candidate SNPs were considered significant if they survived FDR correction based on the number of candidates within each region. In addition to examining the hippocampus and amygdala, for completeness, we examined other subcortical regions at the genome-wide and candidate level. Candidates were selected from GWAS of subcortical volumes, or the GWAS of PTSD. As with the GxE analyses involving PGSs, possible confounding was examined for loci of interest with follow-up models.

## Results

**Fig. 1:**
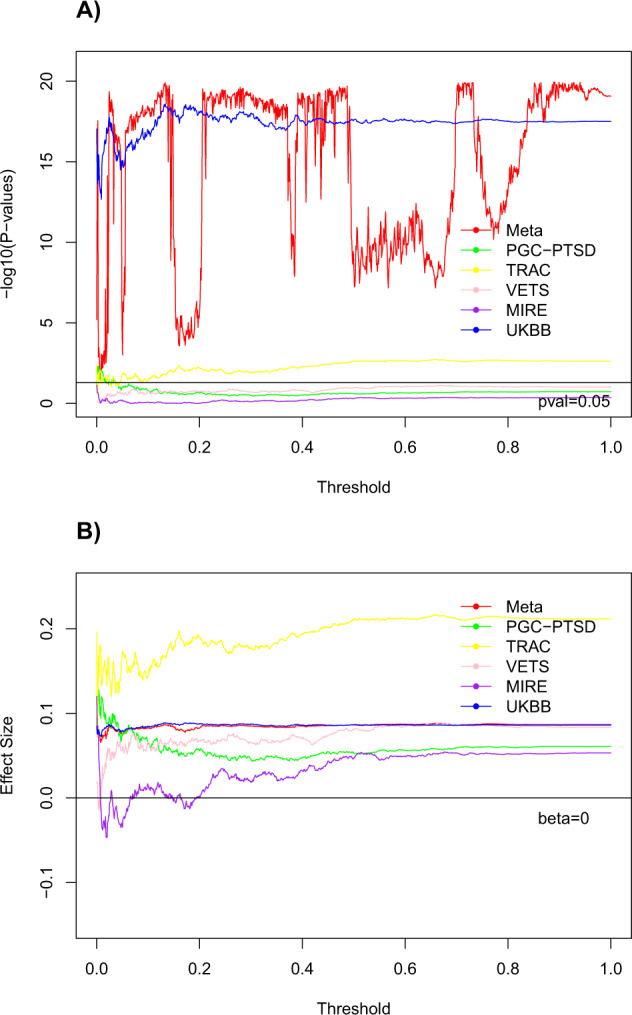
Hibar et al. [26] GWAS hippocampal polygenic score (PGS). The significance (**A**) and effect size estimate (**B**) of the PGS predicting hippocampal volume as a function of the significance threshold. Meta meta-analysis, PGC-PTSD Psychiatric Genetics Consortium-Posttraumatic Stress Disorder, TRAC Translational Research Center for TBI and Stress, VETS Vietnam Era Twin Study of Aging; MIRE MIRE = Duke University and VA Mid-Atlantic Mental Illness Research Education and Clinical Center, UKBB United Kingdom BioBank.

The associations between the hippocampal volume and covariates are provided for each cohort in Supplementary Table 2. We modeled the association between the hippocampal volume and PGS, which were based on the hippocampal ENIGMA-GWAS-2015 and the hippocampal ENIGMA-GWAS-2017 [22, 26]. The hippocampal results for each cohort and the meta-analysis results are presented in Table 2. The most significant hippocampal PGS based on ENIGMA-GWAS-2017 was more strongly associated with hippocampal volume (*p* = 1.10 × 10^−20^) than the most significant PGS based on the ENIGMA-GWAS-2015 (*p* = 6.17 × 10^−8^), and therefore, we used the most significant score based on ENIGMA-GWAS-2017 in subsequent analyses. See Figs. 1A, B for graphs of significance and effect size estimates from hippocampal PGSs at various thresholds based on the ENIGMA-GWAS-2017. The significance of the meta-analysis results is heavily influenced by the association observed in UKBB, and the significance level-shifted substantially depending on whether there was a correspondence between the association observed in UKBB and other cohorts (Fig. 1A). While significance varied, effect size estimates remained remarkably similar across the threshold range, and closely mirrored observations from UKBB (Fig. 1B). In contrast, the amygdala volume PGS, was not significantly associated with amygdala volume (*p* = 0.13).

**Table 2:** Association between hippocampal volume and the polygenic scores for hippocampal volume as computed from the Hibar et al. [22] and the Hibar et al. [26] GWAS at the maximizing thresholds both in the individual cohorts and the meta-analysis across cohorts.

Cohort	GWAS	Threshold	Beta	*p*-value
Meta-analysis	2015	0.0021	0.050	6.17E−08
	2017	0.8891	0.087	1.10E−20
MIRE	2015	0.0001	0.14	0.037
	2017	0.0001	0.12	0.073
PGC-PTSD	2015	0.0001	0.054	0.24
	2017	0.0041	0.13	0.0049
TRAC	2015	0.0361	0.21	0.0033
	2017	0.6591	0.22	0.0018
UKBB	2015	0.1171	0.073	1.25E−13
	2017	0.1331	0.089	2.65E−19
VETS	2015	0.3021	0.10	0.044
	2017	0.6691	0.089	0.076

*PGC-PTSD* Psychiatric Genetics Consortium-Posttraumatic Stress Disorder, *TRAC* Translational Research Center for TBI and Stress, *VETS* Vietnam Era Twin Study of Aging, *MIRE* Duke University and VA Mid-Atlantic Mental Illness Research Education and Clinical Center, *UKBB* United Kingdom BioBank.

The maximizing threshold and associations for the other five subcortical regional PGSs and their corresponding volumes are presented in Supplementary Table 3. For all but one of these, the PGS at the maximizing threshold was significantly associated with the corresponding subcortical volume and survived Bonferroni multiple-testing correction for 1001 thresholds examined (*p* < 5 × 10^−5^). The exception was the pallidum volume PGS, which was nominally associated with pallidum volume at the most significant threshold (*p* = 0.0069) The significance and effect sizes for the association between PGS and volumes as a function of threshold are presented in Supplementary Figs. 1–9. Subsequent analyses of volume PGSs for each subcortical structure used the most significant threshold for that structure.

### Impact of subcortical genetics on PTSD

Next, we tested for main-effect associations between PGS (at the maximizing threshold) and PTSD in each cohort. Although we previously identified association with hippocampal volume and PTSD, both overall and in a follow-up paper, which identified association particularly within those with PTSD and comorbid depression [48], we did not find a significant association between hippocampal PGS and PTSD or hippocampal PGS and childhood trauma (all *p*-values > 0.08). Analysis of amygdala PGS yielded nominally significant associations between the PGS for amygdala volume and PTSD (OR = 1.15, *p* = 0.011). However, the significance of this association is unclear given that the amygdala PGS was not significantly associated with the amygdala volume in our sample (Supplementary Table 3). The other subcortical PGSs were not associated with PTSD (*p* > 0.05). The associations of all subcortical volume PGSs with PTSD are presented in Supplementary Table 4.

### Gene × Environment interaction

**Fig. 2:**
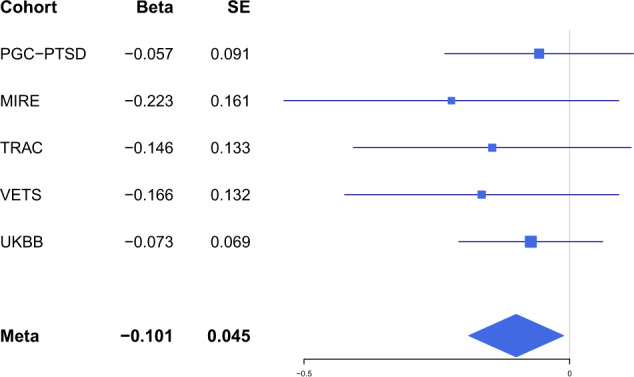
Forest plots of the effect of the GxE interaction between hippocampal polygenic score (PGS) and PTSD its effect on hippocampal volume. Both the PGS and volumes have been standardized (mean = 0, SD = 1). (Meta meta-analysis, PGC-PTSD Psychiatric Genetics Consortium-Posttraumatic Stress Disorder, TRAC Translational Research Center for TBI and Stress, VETS Vietnam Era Twin Study of Aging; MIRE MIRE = Duke University and VA Mid-Atlantic Mental Illness Research Education and Clinical Center, UKBB United Kingdom BioBank).

**Fig. 3:**
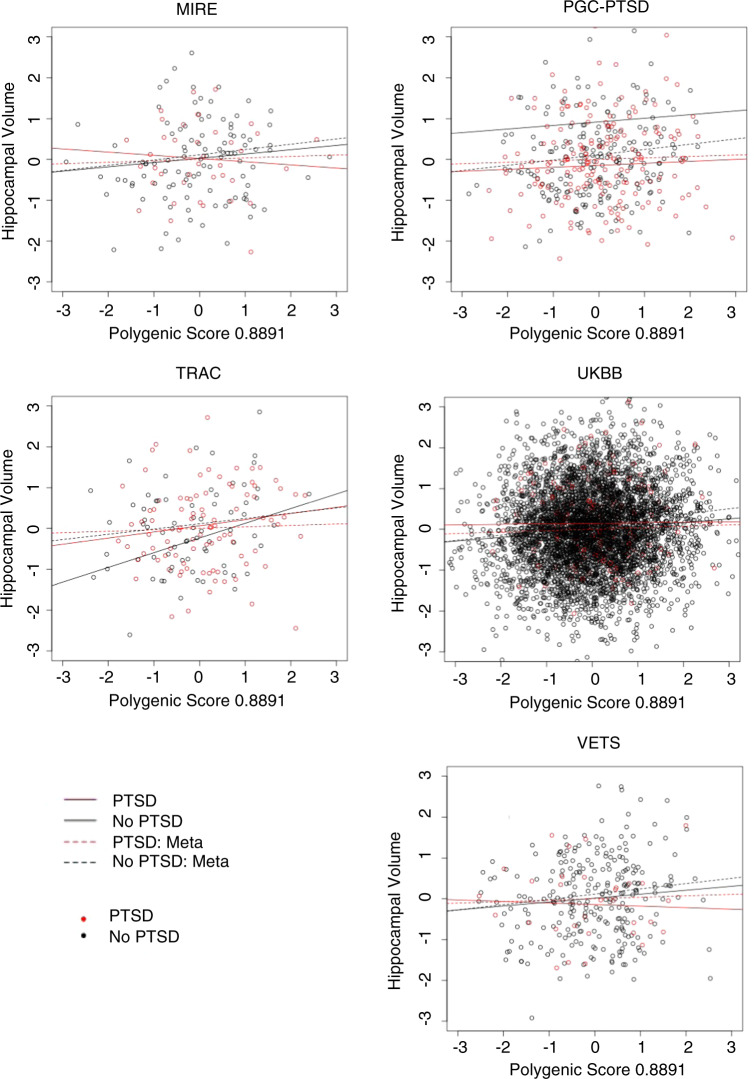
Hippocampal volume as a function of polygenic scores (PGS) with models for PTSD cases and controls in each cohort as well as the meta-analysis model. (Meta meta-analysis, PGC-PTSD Psychiatric Genetics Consortium-Posttraumatic Stress Disorder, TRAC Translational Research Center for TBI and Stress, VETS Vietnam Era Twin Study of Aging; MIRE MIRE = Duke University and VA Mid-Atlantic Mental Illness Research Education and Clinical Center, UKBB United Kingdom BioBank).

Third, we tested for GxE interactions on hippocampal volume. The interaction effect of hippocampal volume PGS (G) and PTSD (E), as well the interaction of hippocampal volume PGS (G) and childhood trauma (E) on hippocampal volume were tested. We observed a significant interaction between PTSD and PGS in predicting hippocampal volume (*beta* = −0.10, *p* = 0.027), indicating that the positive association between the standardized hippocampal PGS and hippocampal volume was reduced, if not eliminated, for subjects with PTSD, although this would not survive a correction for the two interaction terms (PTSD and CT) examined. A forest plot of the PTSD interaction term indicated that the effect was consistently negative across all cohorts (Fig. 2). The fitted model within and across cohorts, with separate predicted hippocampal volumes for cases and controls is presented in Fig. 3. While the pattern of association within each cohort (solid lines) varied, they all demonstrated significant positive associations between PGS and hippocampal volume in controls, and weaker positive (PGC-PTSD, TRAC, UKBB), or negative (VETS, MIRE) associations between PGS and hippocampal volume within PTSD cases. Thus, the meta-analysis (dashed lines) revealed a significant positive association between PGS and hippocampal volume for trauma-exposed controls but no association between PGS and hippocampal volume in PTSD cases.

As the hippocampal volume PGS was nearly as significant across a wide range of thresholds (Fig. 1A), we examined five local peak thresholds (0.024, 0.133, 0.487, 0.730, and 0.889), corresponding to five thresholds where the association between PGS and hippocampal volume was significant (*p* < 10^−19^) for evidence of an interaction with PTSD in follow-up analyses. Most PGSs that we examined had significant PTSD × PGS interactions, where the threshold of 0.487 was the most significant (*p* = 0.0096). While the significance of this GxE interaction was not substantially greater than the *p* = 0.027 significance at a threshold of 0.889, we note that this significance exceeds a Bonferroni correction for the five thresholds we examined, and this follow-up examination demonstrates that the observed GxE interaction effect is robust in relation to the threshold, given that the GxE effect was significant at all thresholds except one value (threshold = 0.024, *p*_GxE_ = 0.084). Interaction models for the amygdala and the other subcortical volumes did not yield significant GxE effects for either PTSD or childhood trauma. Details are in Supplementary Table 5.

### Gene × Environment GWAS

The GxE GWASs of the amygdala and hippocampus with childhood trauma as the interacting factor not appear to be inflated (lambda = 0.98 and 0.96 respectively), although modest inflation was observed for the GxE GWAS of the amygdala and hippocampus with PTSD as the interacting factor (lambda = 1.077 and 1.16). See Supplementary Figs. 8 and 9 for QQ plots of the GxE associations.

The most significant GxE effect in these regions was an intergenic SNP rs4702973 × childhood trauma interaction (*p* = 2.16 × 10^−7^) associated with amygdala volume. The effect of this SNP was consistent across cohorts (Supplementary Fig. 10). Interestingly, among the top hits, a PTSD × rs10861272 interaction was associated with amygdala volume. This SNP is in the gene *CHST11* which was recently implicated in a military epigenome-wide association study of PTSD [49]. However, no genome-wide significant interactions were observed with PTSD or childhood trauma in either brain region. See Supplementary Table 6A for the top GxE associations in the hippocampus and amygdala.

We then examined the candidate variants. Six interactions were nominally significantly associated with either amygdala or hippocampal volume (Supplementary Table 7A), although none survived correction. The most significant candidate interaction observed was at rs148757321 × childhood trauma interaction that was associated with hippocampal volume (*p* = 0.0030). The rs148757321 SNP in the *KAZN* gene has previously been implicated in a GWAS of PTSD [27].

There was no evidence of inflation for the GxE GWAS of other subcortical regions (Supplementary Fig. 8). We found no genome-wide significant associations. The most significant GxE association was observed between an intergenic SNP rs2348408 with PTSD (beta = −7.5394, *p* = 1.471 × 10^−7^) in association with nucleus accumbens volume. See Supplementary Table 6B for the top GxE associations outside of the hippocampus and amygdala. Candidate variant GxE analyses of the other subcortical regions did not produce any significant associations following correction. The top GxE associations outside of the hippocampus and amygdala are summarized in Supplementary Table 7B.

### Examination of potential confounding effects

We then explored the possibility that PGS × PTSD effects in the hippocampus and rs4702973 × CT effect in the amygdala were due to confounding with sex, age, or population substructure. Note that the pattern of results observed in our cohorts (Fig. 2 for hippocampus, Supplementary Fig. 10 for amygdala) do not support a spurious interaction related to sex or age, as the pattern of association is similar across cohorts despite demographic differences (e.g. only males in the VETSA cohort; Table 1). The GxE GWAS results do not support spurious interactions due to cryptic population substructure within European-descent participants examined here, as this would likely lead to widespread inflation in the GxE GWAS, which was not observed (Supplementary Figs. 9 and 10). Next, there was little evidence of subcortical volume-associated substructure except possibly in UKBB (Supplementary Table 2), and observed PGSs are not strongly associated with PTSD. Finally, we ran a series of models in UKBB (the largest cohort) including interaction terms involving age, sex, and volume-associated PCs. We note the PGS × PTSD effect in the hippocampus and rs4702973 × CT effect in the amygdala did not change substantially when these terms were added (Supplementary Table 8). Therefore, confounding with these three factors is an unlikely explanation for our GxE effects. We are not able to examine possible effects of confounding with smoking, alcohol, drug use, or PTSD treatment, as these data were generally not available across cohorts.

### Imbalance of cases and controls

The low percentage of cases (10.6%; Table 1) was a result of samples from UKBB, which contributed many more controls (*n* = 6410) than cases (*n* = 160). To address our concerns about this imbalance, we conducted additional testing to determine whether including UKBB was detrimental to our analyses or altered our findings, but found no detrimental effect of including UKBB data.

## Discussion

In this study, we derived polygenic scores (PGS) that capture the influence of genetic variants on the volume of the hippocampus, amygdala, and other subcortical structures using two previous GWAS in discovery samples reported by the ENIGMA Consortium [22, 26]. We found that subcortical volume PGSs were strongly associated with respective subcortical volumes for the hippocampus, thalamus, caudate, putamen, and nucleus accumbens, which were consistent with the two discoveries GWAS [22, 26]. Most notably, we found a significant association between the hippocampal volume PGS and hippocampal volume in control subjects that were absent in individuals with PTSD (GxE). Indeed, this significant GxE relationship persisted in four out of five threshold peaks that were used to calculate hippocampal volume PGSs from published hippocampal GWAS. We did not find similar GxE interactions with exposure to childhood trauma or with PTSD in other subcortical regions.

The interaction of hippocampal volume PGS and PTSD on hippocampal volume warrants deeper consideration. Among controls, we found a positive association of hippocampal volume PGS and hippocampal volume. This result is consistent with the meta-analysis result in our full sample of cases and controls that demonstrated an association between hippocampal volume PGS and hippocampal volume, and as expected, was consistent with ENIGMA-GWAS-2017 [26]. It is possible that the lack of an association among PTSD cases in the PGC-PTSD, UKBB, and TRAC cohorts, and the negative association among PTSD cases in the VETS and MIRE cohorts may be due to an atrophic influence of trauma and PTSD on hippocampal volume [6, 50]. Whereas PGS is linked with volume in healthy environments, exposure to trauma and onset of PTSD may have reduced this genetic influence on hippocampal volume. An open question remains whether trauma, PTSD, or trauma + PTSD served as mitigating factors. Most of our cohorts had childhood trauma information and many lacked adult trauma exposure metrics, which hindered deeper investigation of the role of trauma vis-à-vis PTSD. In the trauma field broadly, PTSD diagnosis/severity and trauma exposure are highly collinear and individuals with PTSD experience significantly more trauma than trauma-exposed controls, which has posed an ongoing challenge to investigating the relative contributions of trauma and PTSD on various outcomes. Relatedly, we examined the interaction effect of hippocampal volume PGS and childhood trauma on hippocampal volume, which was not significant. It is possible that the lack of an interaction effect with childhood trauma may be explained by trauma exposure that was sufficiently remote compared to more recent PTSD symptomatology.

We observed an association between the PGS for amygdala volume and PTSD. This is an interesting finding, which offers evidence that smaller amygdala volume, or at least biological processes underlying the genetic architecture of the amygdala are causally related to PTSD. The amygdala response to trauma and stress stands in clear contrast to the response of the hippocampus [50–52]. The hippocampus undergoes dendritic atrophy and debranching of pyramidal neurons whereas amygdala pyramidal and stellate neurons exhibit enhanced dendritic arborization in rats [50–52]. Thus, the balance of phenotypic variance from amygdala volume PGS relative to the environment is altered under conditions of trauma exposure and stress. This supports animal-model results linking amygdala volume and PTSD risk. In mice, a small basolateral amygdala volume predicts fear, anxiety, and stress-related behaviors [53].

Thus, it is also possible that humans who have a PGS which is associated with a smaller amygdala are more vulnerable to developing PTSD following trauma or chronic stress. On the other hand, larger amygdala volume is linked to greater risk-taking behaviors [54], which may increase the risk of trauma exposure and in turn the risk of developing PTSD [55]. The size of the basolateral amygdala in the genetically selected mice also predicts locomotor exploration in novel environments. One hypothesis supported by our results that will require independent confirmation, is whether behavior such as greater exploration of novel environments, which is linked to basolateral amygdala volume and is genotypically conferred, may place children at greater risk of experiencing trauma. This hypothesis presupposes that behaviors promoting exploration of novel environments incurs an elevated risk of exposure to childhood trauma. On the contrary, there is also evidence that novelty inhibition poses a greater risk of PTSD and novelty seeking is associated with reduced risk of PTSD [56]. Nonetheless, several caveats are in order. The first is that associative fear learning is a representative model system for PTSD [15, 16, 57]. The second is that linking specific genotypic profiles with behaviors that may increase the risk of trauma exposure or PTSD is intended to deflect, rather than to engage, in victim-blaming.

The SNP × childhood trauma interaction effect on amygdala volume was found for the intergenic marker rs4702973 located between the *LOC285706* and *EEF1A1P20* genes. Very little is known about the role of this region on chromosome 5. A SNP in this region has been associated with acute lymphoblastic leukemia (ALL) that most commonly occurs in childhood and teens. However, ascribing this region as vulnerable to the effects of childhood trauma or stress is speculative as the only known environmental risk factors for developing ALL are exposure to radiation and chemical agents. Proximity to *EEF1A1P20* is non-informative given it is a designated pseudogene, which are non-functional copies of functional genes. The SNP × childhood interaction effect on hippocampal volume is with rs148757321, a SNP in the *KAZN* gene. This gene is expressed in the brain [58] at low levels in the glia and neurons of the hippocampus, and encodes a protein involved with desmosome assembly, cell adhesion, and cytoskeletal organization [59]. The *KAZN* gene is associated with PTSD [27] and schizophrenia [60], as well as three neurodegenerative diseases including Alzheimer’s disease, Parkinson’s disease, and amyotrophic lateral sclerosis [59].

Additionally, the association with PTSD was observed with an amygdala PGS that lacked a significant association with amygdala volume, at least with the current sample size. This is perhaps not surprising given the lower heritability for amygdala volume reported in twin studies, the smaller number of GWAS loci observed for amygdala volume in the subcortical volume GWAS, and the lower proportion of variance explained by common (GWAS-detectable) variants. Therefore, it is unclear why a poor predictor of amygdala volume would be a better predictor of PTSD. It is conceivable that the amygdala PGS is acting as an index of underlying biological processes that can lead to both small amygdala volume and risk for PTSD. However, it is unclear, given the small proportion of variance of amygdala volume explained by common variants, why these common variants would accurately represent these biological processes. Perhaps more relevant to the present sample, is the role of environment, particularly trauma exposure and PTSD. Model systems in mice [61] and macaques [62] show hypertrophy of amygdala following severe chronic stress, which has been recapitulated in longitudinal human studies of police before and after periods of occupational trauma and stress [63]. Thus, amygdala hypertrophy due to environmental trauma may be a non-genetic contribution that explains the lack of an association between amygdala volume PGS and amygdala volume in our sample, but not in the trauma-exposed normative ENIGMA-GWAS-2015. Therefore, we must treat this association between the amygdala PGS and PTSD as provisional. Further verification of this result is required, and this is likely to come in the form of more sophisticated models of causality such as Mendelian Randomization which will be enabled once suitably large sample GWASs of subcortical volume, PTSD, and trauma are available.

### Limitations and strengths

Several limitations of our study deserve mention. Chiefly, the uneven availability of covariates across sites precluded a detailed examination of several environmental factors that may interact with subcortical volume PGSs to influence subcortical volume such as socioeconomic status, education level, poverty, chronic stress, alcohol use, premature birth, exercise/fitness, comorbid illness, trauma chronicity, and treatment history. In particular, metrics for PTSD chronicity and treatment duration as well as trauma chronicity and severity may have improved our models, and to some extent, their absence limits our findings. Whereas the PGS approach requires fewer subjects than GWAS, our study was nonetheless underpowered to detect small effect sizes. Trauma exposure—particularly childhood trauma—relies on the recall of distant memories and may therefore be susceptible to underreporting [64, 65]. In the future, we may apply PGS derived broadly from discovery samples with genetically correlated psychiatric disorders such as depression, schizophrenia, etc., to test in our target sample of trauma and PTSD. Finally, our results would not survive corrected significance testing if corrected across all subcortical volumes. However, we had a priori hypotheses concerning the hippocampus and amygdala supported by prior work documented by preregistration of hypotheses in our research proposal (https://pgc-ptsd.com/wp-content/uploads/2020/12/3996157_Egrant_Salaries_Redacted.pdf). Lastly, we were unable to calculate separate PGS of left and right hemisphere structures are given that GWAS by Hibar et al. [22, 26] did not report separate statistics for left and right hemisphere structures, and this will be an important area for further study.

Our study has several strengths. It included data from a diverse set of cohorts with respect to age, gender, geography, and trauma type. The relative effect sizes associated with the PTSDxPGS interaction on hippocampal volume and rs4702973xCT interaction on amygdala volume are consistent across cohorts (Fig. 2 and Supplementary Fig. 10). Relative contributions of covariates, which included age, age [2], sex, and PCs, were generally consistent across the cohorts we studied (Supplementary Table 2). Each cohort used different MRI scanner manufacturers and different acquisition sequences, which may contribute to heterogeneity across sites. However, such a conclusion is not supported by our published meta-regression results, which demonstrated that subcortical volume effect size estimates for cohorts did not vary as a function of cohort age, the proportion of females, level of childhood trauma exposure, scanner strength, and FreeSurfer version [4]. Finally, regarding novelty, many published studies have (1) examined the effects of genes on subcortical volume in normative samples [22, 26], (2) examined the role of PTSD/trauma on subcortical volumes [4, 5], and (3) polygenic risk scores from GWAS of various neuropsychiatric disorders have been applied to examine the role of genetic variants on brain and cognition [66–69], very little has been published about genetic and environmental interactions on subcortical volumes [70].

## Conclusion

Both the hippocampus and amygdala are pivotal brain structures in mediating PTSD symptomatology. In this study, we find evidence that trauma exposure and PTSD modulate the effect of polygenic markers on hippocampal volume (GxE) and that the amygdala volume PGS itself is associated with PTSD risk, which supports the role of amygdala volume as a risk factor for PTSD.

## Supplementary information

Supplemental Materials
