# Supplementary material for: Trauma and posttraumatic stress disorder modulate polygenic predictors of hippocampal and amygdala volume
Source: Transl Psychiatry. 2021 Dec 16;11:637. doi: 10.1038/s41398-021-01707-x (PMC8677780; doi:10.1038/s41398-021-01707-x)
Supplement: Supplementary file 1 — Supplemental Materials [file 41398_2021_1707_MOESM1_ESM.pdf]

Supplementary Materials

Supplementary Methods

In addition to examining the dichotomous PTSD diagnosis variable, we examined a PTSD severity (PTSDsev) variable. For almost every cohort, this was assessed using the CAPS or PCL (see Supplementary Table 1 for details). The stage 2 and stage 3 models were very similar to those fit for PTSD. That is, in stage 2, linear models were fit with PTSDsev as the response, and the same set of predictors. A mixed model was used for VETSA to allow for correlation within twin pairs. In Stage 3, the main effect for PTSDsev and a PTSDsev x PGS interaction were added to the models of subcortical volume.

Supplementary Results.

The Stage 2 PTSDsev analyses yielded similar results to the dichotomous PTSD analysis. That is, the PGS for hippocampal volume was not associated with PTSDsev. When examining other cortical regions, only the amygdala PGS was associated with PTSDsev (beta=-0.27, p=0.036), with significance similar to that observed with PTSD (OR=1.15, p=0.011). The stage 2, the interaction effect of PTSDsev and hippocampal volume PGS negative effect, as estimated for PTSD, but only at the trend level (p=0.084). For all other subcortical volumes, the interaction of PTSDsev and PGS was not significant (p>0.10).

Supplementary Figure 1: Accumbens PGS: The significance (**A**) and effect size estimate (**B**) of the PGS predicting accumbens volume as a function of the threshold.

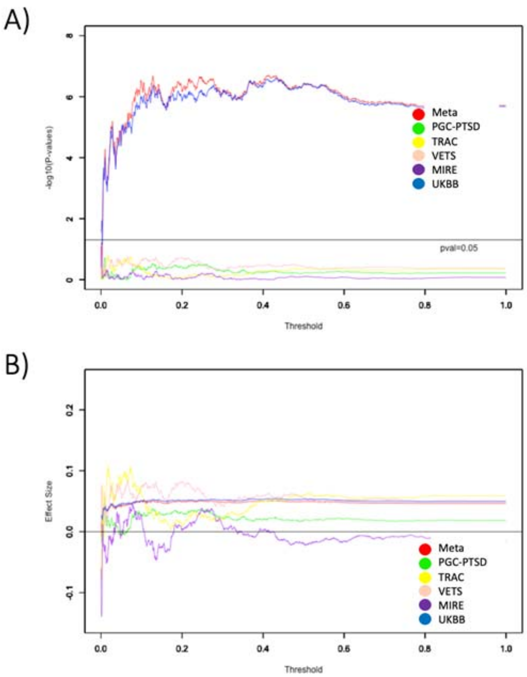

Supplementary Figure 2: Amygdala PGS: The significance (**A**) and effect size estimate (**B**) of the PGS predicting amygdala volume as a function of the threshold.

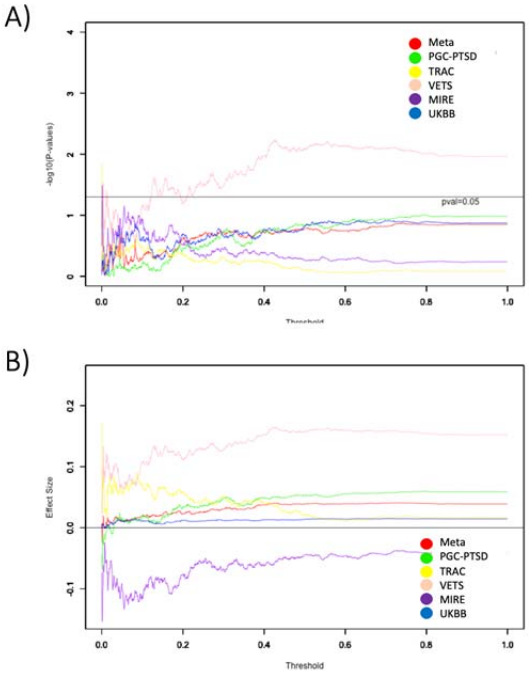

Supplementary Figure 3: Caudate PGS: The significance (**A**) and effect size estimate (**B**) of the PGS predicting caudate volume as a function of the threshold.

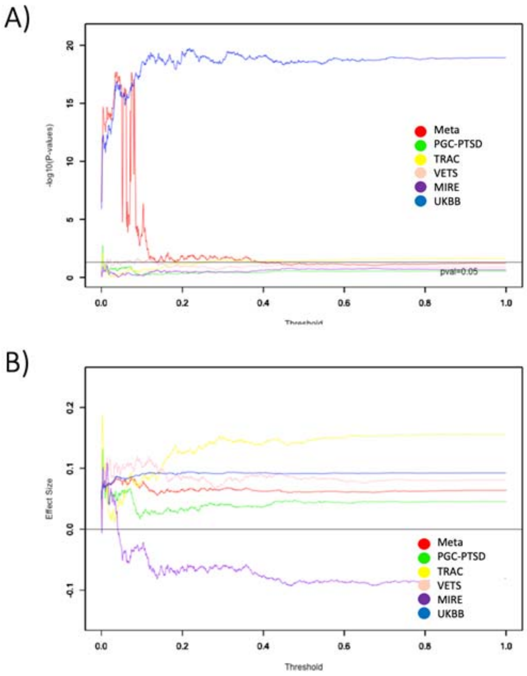

Supplementary Figure 4 – Hibar et al. 2015 GWAS Hippocampal PGS: The significance (**A**) and effect size estimate (**B**) of the PGS predicting hippocampal volume as a function of the threshold.

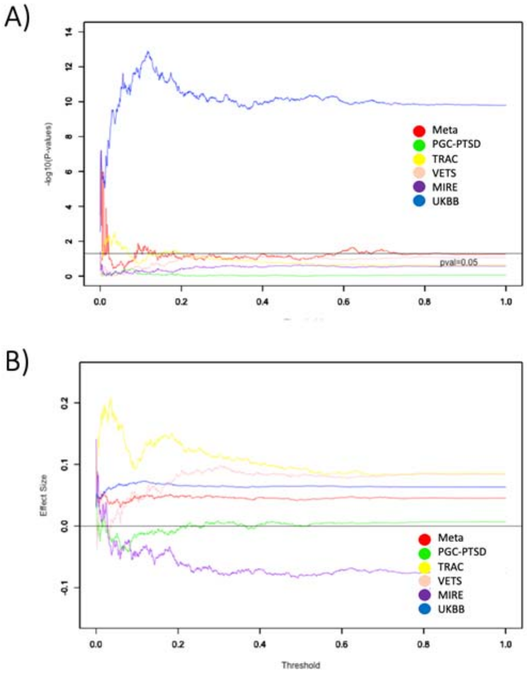

Supplementary Figure 5 –Pallidum PGS: The significance (**A**) and effect size estimate (**B**) of the PGS predicting pallidum volume as a function of the threshold.

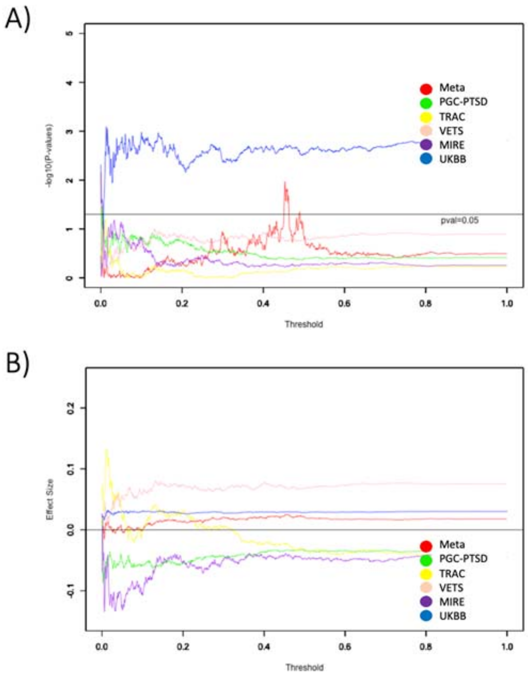

Supplementary Figure 6 –Putamen PGS: The significance (**A**) and effect size estimate (**B**) of the PGS predicting putamen volume as a function of the threshold.

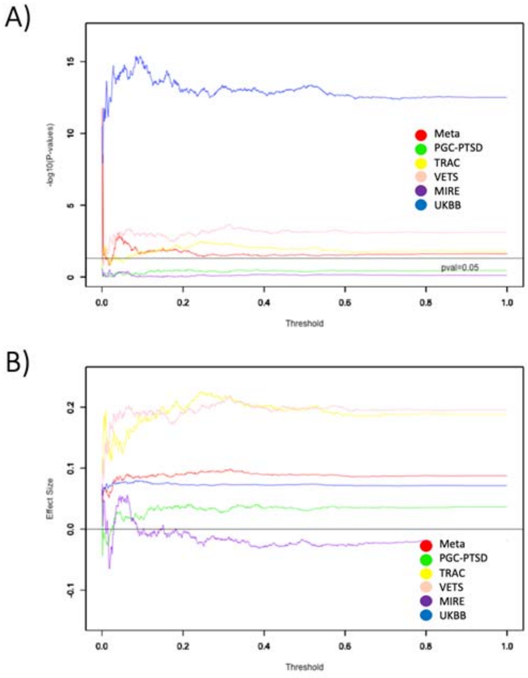

Supplementary Figure 7 –Thalamus PGS: The significance (**A**) and effect size estimate (**B**) of the PGS predicting thalamus volume as a function of the threshold.

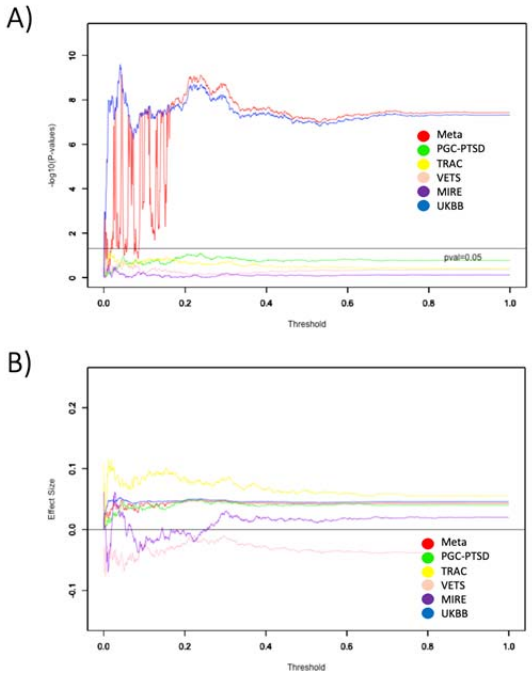

Supplementary Figure 8: QQ plots of the GxE GWASs of PTSD predicting subcortical volumes.

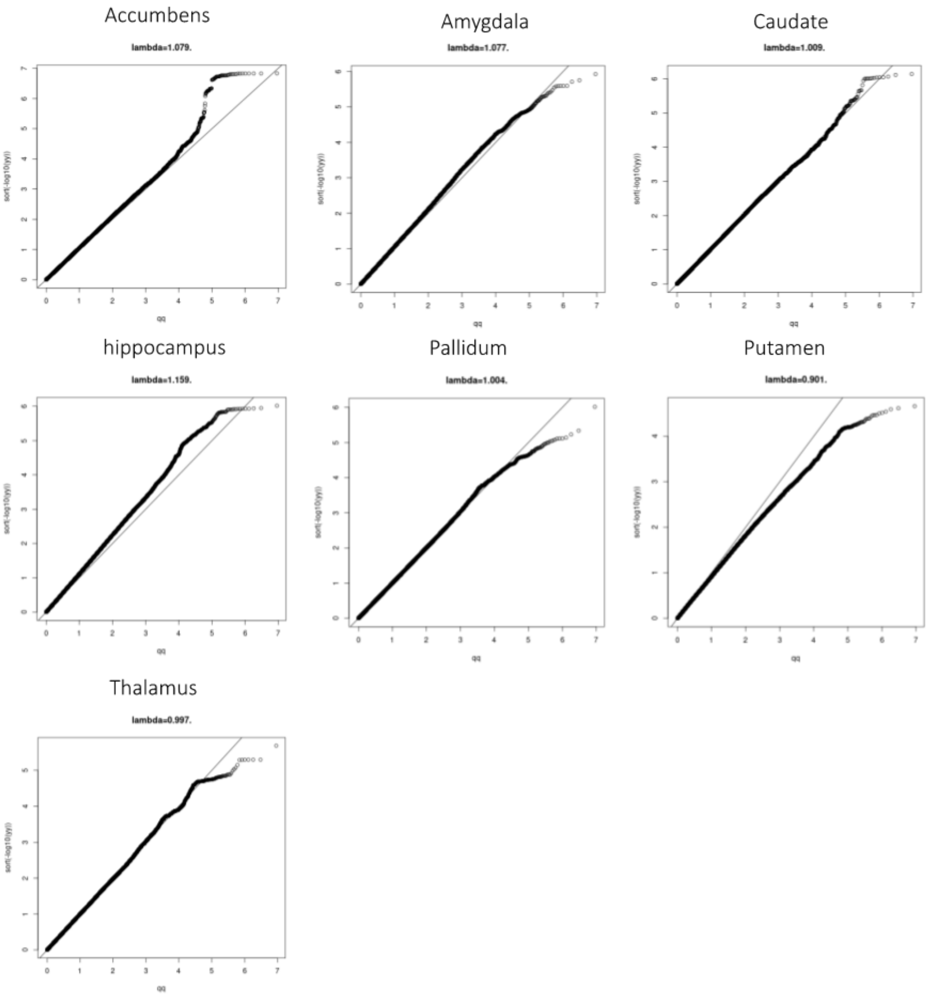

Supplementary Figure 9: QQ plots of the GxE GWAS of childhood trauma predicting subcortical volumes.

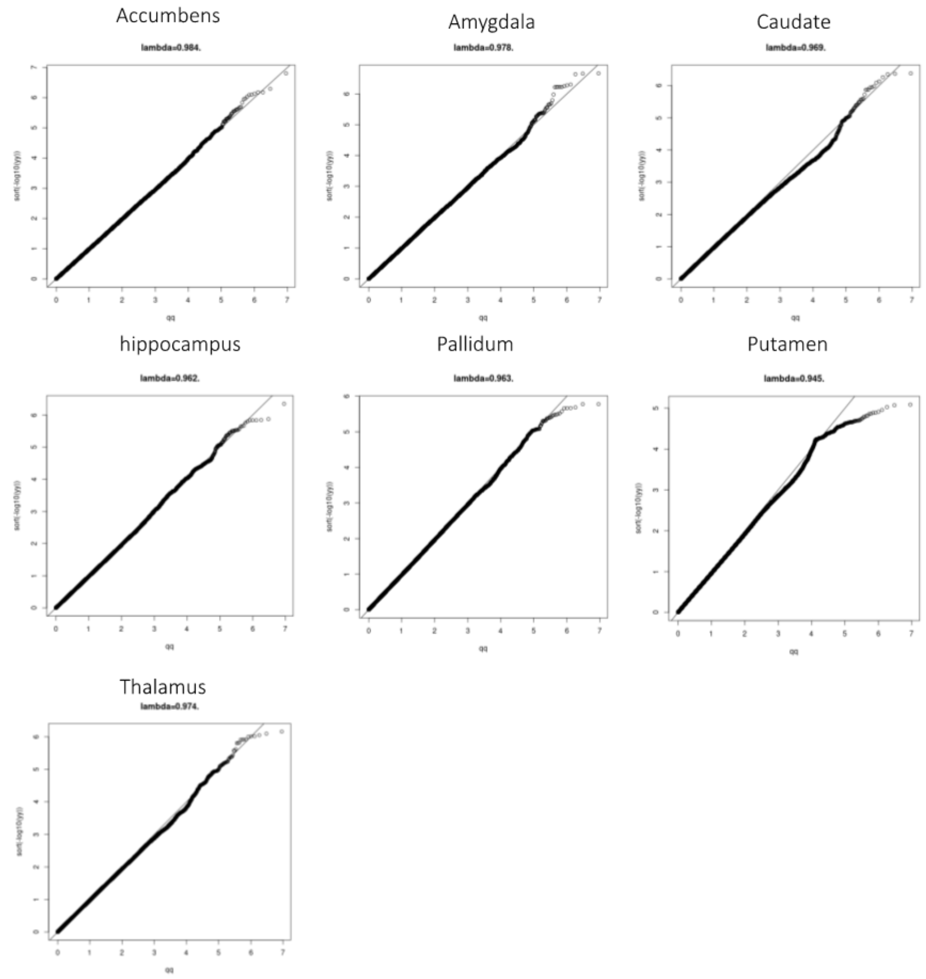

Supplementary Figure 10: Forest plots of the effect of the GxE interaction between rs4702973 and CT on amygdala volume. Both volume has been standardized (mean=0, SD=1). (Abbreviations: Meta=meta-analysis, PGC-PTSD=Psychiatric Genetics Consortium-Posttraumatic Stress Disorder, TRAC=Translational Research Center for TBI and Stress, VETS=Vietnam Era Twin Study of Aging; MIRE= MIRE=Duke University and VA Mid-Atlantic Mental Illness Research Education and Clinical Center, UKBB=United Kingdom BioBank).

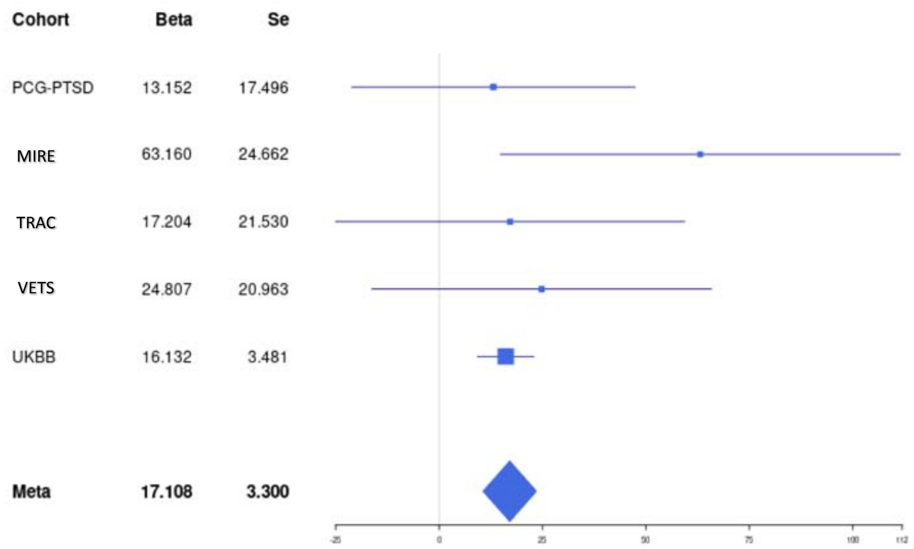

Supplementary Table 1: Methods for assessing subcortical volume, genotypes, and PTSD and Childhood Trauma (CT) across included cohorts.

Cohort (sub-cohort)	Scanner/ Software	Genotyping Platform	Imputation Method/Panel	European Ancestry criterion	Additional Cleaning	PTSD Severity Assessment	PTSD (case/ control) Criterion	CT Assessment
PGC-PTSD								
(BETE)	Philips Achieva	Illumina Human OmniExpress 24 v1.1	IMPUTE2/1000 Genomes Phase 3	SNPweights	Call rate>95%, removed individuals with sex discordance, high inbreeding coefficients, high relatedness	CAPS 4	CAPS>45/DSM 4 criteria	ETI
(DEFE)	Siemens TimTrio	Illumina PsychChip	IMPUTE2/1000 Genomes Phase 3	SNPweights	Call rate>95%, removed individuals with sex discordance, high inbreeding coefficients, high relatedness	CAPS 4	DSM criteria	n/a
(BRY2)	GE Signa	Illumina PsychArray	IMPUTE2/1000 Genomes Phase 3	SNPweights	Call rate>95%, removed individuals with sex discordance, high inbreeding coefficients, high relatedness	CAPS 4	DSM criteria	n/a
(KMCT)	Philips Achieva/ Siemens Tim Trio	Illumina PsychArray	IMPUTE2/1000 Genomes Phase 3	SNPweights	Call rate>95%, removed individuals with sex discordance, high inbreeding coefficients, high relatedness	UCLA	DSM 5 criteria (highest of parent or child)	CTQ
TRAC	Siemens Tim Trio / Freesurfer 5.1	Illumina HumanOmni2.5-8	IMPUTE2/1000 Genomes Phase 1	SNPweights	Call rate>95%, removed sex discordance	Diagnostic interview	DSM 4 criteria	TLEQ
VETS	Siemens TimTrio	Illumina HumanOmniExpress-24 v1.0A beadchips	MACH/ 1000 Genomes Phase 3	SNPweights and PCA	Twin relationships confirmed	PCL-C	DSM 3 criteria	Adapted Holmes-Rahe
MIRE	GE MR750	Illumina HumanHap650 beadchips/ Illumina Human1M-Duo beadchips/ Illumina HumanOmni2.5 beadhips	IMPUTE2/1000 Genomes	SNPweights and PCA	Call rate>98%, removed duplicate and related individuals, removed sex discordance	DTS	SCID for DSM 4	CTQ
UKBB	Siemens Skyra	Affymetrix Axiom	HRC/UK10K	4-means clustering	Removed related individuals, high level of missingness or heterozygosity, gender discordance	Adapted PCL-S and PHQ9	Cutoff score	Adapted CTQ

Abbreviations: DEFE= Defining Essential Feature of Neural Damage - VA Minneapolis HealthCare System, Minneapolis MN USA; PGC-PTSD=Psychiatric Genetics Consortium-Posttraumatic Stress Disorder; TRAC= Translational Research Center for TBI and Stress, Boston VA HealthCare System, Boston MA USA; VETS=Vietnam Era Twin Study of Aging, San Diego VA Healthcare System, San Diego CA USA; MIRE=Duke University and VA Mid-Atlantic Mental Illness Research Education and Clinical Center the Study of Post-Deployment Mental Health Study, Durham NC USA; UKBB=United Kingdom BioBank; BRY2=Bryant2 Sydney Neuroimaging, University of New South Wales Australia, KMCT=Katie McLaughlin Child Trauma, Child Trauma and Neural Systems Underlying Emotion Regulation, University of Washington, Seattle WA USA; BETR= Biological Effects of Traumatic Experiences, University Medical Center, Utrecht Netherlands

Supplementary Table 2 (A to G): Significance of the model covariates in predicting subcortical volumes

A) Accumbens

Cohort	Covariate	Beta	P-Value
**MIRE**	(Intercept)	-0.12	0.61
	Age	0.24	0.67
	Age^2	-0.37	0.52
	Gender	0.13	0.59
	ICV	0.52	1.82E-10
	PC1	0.0038	0.96
	PC2	-0.012	0.87
	PC3	0.036	0.62
	PC4	0.064	0.37
**PGC-PTSD**	(Intercept)	1.94	8.77E-24
	Age	-0.27	0.32
	Age^2	0.05	0.85
	Gender	-0.1	0.39
	ICV	0.46	1.76E-15
	PC1	0.097	0.0087
	PC2	-0.014	0.7
	PC3	0.018	0.62
	PC4	0.026	0.48
	siteMinneapolis	-2.32	1.98E-44
	siteNSW	-2.26	9.20E-40
	siteU Wash	-2.2	6.25E-14
**TRAC**	(Intercept)	-0.062	0.85
	Age	-0.32	0.55
	Age^2	0.078	0.89
	Gender	0.067	0.84
	ICV	0.29	0.0006
	PC1	-0.018	0.81
	PC2	0.019	0.8
	PC3	0.077	0.3
	PC4	0.035	0.64
**UKBB**	(Intercept)	-0.082	2.03E-07

	Age	0.28	0.06
	Age^2	-0.61	4.36E-05
	Gender	0.17	7.28E-12
	ICV	0.37	1.17E-176
	PC1	0.0042	0.69
	PC10	-0.0022	0.84
	PC2	0.011	0.3
	PC3	-0.012	0.23
	PC4	0.026	0.013
	PC5	-8.00E-05	0.99
	PC6	-0.013	0.2
	PC7	0.018	0.091
	PC8	0.0011	0.92
	PC9	-0.014	0.19
**VETS**	(Intercept)	-0.018	0.79
	Age	-0.063	0.49
	Age^2	-0.053	0.56
	ICV	0.16	0.0092
	PC1	0.0088	0.91
	PC2	0.0065	0.93
	PC3	0.055	0.45
	PC4	-0.0066	0.93

B) Amygdala

Cohort	Covariate	Beta	P-Value
**MIRE**	(Intercept)	-0.34	0.13
	Age	-0.42	0.45
	Age^2	0.38	0.5
	Gender	0.38	0.11
	ICV	0.52	1.34E-10
	PC1	-0.11	0.14
	PC2	0.0083	0.91
	PC3	-0.037	0.6
	PC4	0.072	0.31
**PGC-PTSD**	(Intercept)	1.62	1.71E-17
	Age	-0.13	0.64
	Age^2	0.022	0.93

	Gender	0.25	0.034
	ICV	0.39	8.21E-12
	PC1	0.0031	0.93
	PC2	-0.036	0.34
	PC3	0.0063	0.86
	PC4	0.042	0.25
	siteMinneapolis	-2.34	1.27E-44
	siteNSW	-2.05	7.02E-34
	siteU Wash	-2.49	5.88E-17
**TRAC**	(Intercept)	-0.0081	0.98
	Age	-0.11	0.84
	Age^2	0.033	0.95
	Gender	0.0086	0.98
	ICV	0.45	5.44E-08
	PC1	-0.023	0.75
	PC2	-0.001	0.99
	PC3	0.032	0.66
	PC4	0.14	0.046
**UKBB**	(Intercept)	-0.15	3.65E-24
	Age	0.71	2.14E-07
	Age^2	-1	2.28E-13
	Gender	0.31	3.43E-41
	ICV	0.47	<1E-176
	PC1	-0.0034	0.72
	PC10	0.002	0.83
	PC2	-0.008	0.4
	PC3	-0.0027	0.78
	PC4	0.01	0.28
	PC5	0.012	0.22
	PC6	-0.02	0.032
	PC7	0.014	0.15
	PC8	-0.0028	0.77
	PC9	-0.0034	0.72
**VETS**	(Intercept)	-0.016	0.81
	Age	-0.037	0.68
	Age^2	-0.02	0.82
	ICV	0.25	3.38E-05

	PC1	-0.003	0.97
	PC2	-0.0021	0.98
	PC3	-0.0081	0.91
	PC4	0.0084	0.9

C) Caudate

Cohort	Covariate	Beta	P-Value
**MIRE**	(Intercept)	-0.0096	0.96
	Age	-0.74	0.14
	Age^2	0.44	0.38
	Gender	0.011	0.96
	ICV	0.56	7.51E-14
	PC1	-0.093	0.16
	PC2	0.021	0.74
	PC3	0.016	0.8
	PC4	0.15	0.022
**PGC-PTSD**	(Intercept)	0.94	1.28E-05
	Age	-1.13	0.00049
	Age^2	0.89	0.0043
	Gender	-0.042	0.76
	ICV	0.75	4.16E-26
	PC1	0.063	0.15
	PC2	-0.039	0.37
	PC3	0.065	0.12
	PC4	0.056	0.2
	siteMinneapolis	-1.29	2.84E-13
	siteNSW	-0.78	1.87E-05
	siteU Wash	-1.69	6.62E-07
**TRAC**	(Intercept)	0.012	0.97
	Age	-1.25	0.015
	Age^2	0.95	0.061
	Gender	-0.013	0.97
	ICV	0.37	3.41E-06
	PC1	0.064	0.36
	PC2	0.023	0.73
	PC3	0.021	0.76

	PC4	-0.079	0.26
**UKBB**	(Intercept)	0.013	0.41
	Age	-0.29	0.046
	Age^2	0.28	0.05
	Gender	-0.029	0.24
	ICV	0.58	<1E-176
	PC1	0.0089	0.38
	PC10	0.0049	0.63
	PC2	0.0073	0.47
	PC3	0.014	0.17
	PC4	0.0077	0.44
	PC5	-0.0052	0.6
	PC6	-0.0078	0.44
	PC7	0.0068	0.5
	PC8	0.01	0.3
	PC9	-0.0081	0.42
**VETS**	(Intercept)	-0.0071	0.9
	Age	0.064	0.43
	Age^2	0.013	0.87
	ICV	0.51	1.19E-18
	PC1	0.016	0.8
	PC2	-0.057	0.36
	PC3	0.032	0.6
	PC4	-0.0021	0.97

D) Hippocampus

Cohort	Covariate	Beta	P-Value
**MIRE**	(Intercept)	0.054	0.8
	Age	0.52	0.33
	Age^2	-0.58	0.27
	Gender	-0.061	0.79
	ICV	0.59	6.06E-14
	PC1	0.071	0.31
	PC2	0.019	0.78
	PC3	0.11	0.11
	PC4	0.11	0.095
**PGC-PTSD**	(Intercept)	0.35	0.13

	Age	-0.42	0.23
	Age^2	0.22	0.5
	Gender	0.24	0.11
	ICV	0.56	2.17E-14
	PC1	-0.03	0.52
	PC2	0.0054	0.91
	PC3	0.0091	0.84
	PC4	0.063	0.17
	siteMinneapolis	-1.05	2.29E-08
	siteNSW	0.038	0.84
	siteU Wash	-1.48	4.38E-05
**TRAC**	(Intercept)	-0.14	0.64
	Age	-0.017	0.97
	Age^2	-0.18	0.72
	Gender	0.15	0.64
	ICV	0.46	1.27E-08
	PC1	0.067	0.34
	PC2	0.073	0.28
	PC3	0.12	0.074
	PC4	0.11	0.11
**UKBB**	(Intercept)	-0.028	0.06
	Age	1.25	1.55E-18
	Age^2	-1.54	5.14E-27
	Gender	0.059	0.014
	ICV	0.5	<1E-176
	PC1	0.012	0.23
	PC10	0.0011	0.91
	PC2	-0.0085	0.39
	PC3	-0.00096	0.92
	PC4	0.016	0.11
	PC5	0.032	0.0011
	PC6	-0.02	0.042
	PC7	-0.00058	0.95
	PC8	-0.004	0.69
	PC9	0.0074	0.46
**VETS**	(Intercept)	0.0079	0.88
	Age	-0.18	0.012

	Age^2	-0.13	0.073
	ICV	0.5	1.10E-19
	PC1	0.14	0.021
	PC2	0.053	0.35
	PC3	-0.11	0.042
	PC4	-0.086	0.13

E) Pallidum

Cohort	Covariate	Beta	P-Value
**MIRE**	(Intercept)	-0.63	0.0069
	Age	-0.42	0.46
	Age^2	0.12	0.84
	Gender	0.71	0.0045
	ICV	0.3	0.00013
	PC1	-0.1	0.17
	PC2	0.012	0.87
	PC3	0.11	0.13
	PC4	0.068	0.35
**PGC-PTSD**	(Intercept)	0.76	0.0002
	Age	-0.51	0.1
	Age^2	0.16	0.6
	Gender	0.34	0.013
	ICV	0.59	6.28E-19
	PC1	0.044	0.29
	PC2	-0.028	0.51
	PC3	0.033	0.41
	PC4	0.048	0.25
	siteMinneapolis	-1.71	1.07E-22
	siteNSW	-0.67	9.99E-05
	siteU Wash	-0.69	0.031
**TRAC**	(Intercept)	-0.22	0.47
	Age	-0.013	0.98
	Age^2	-0.11	0.83
	Gender	0.23	0.46
	ICV	0.44	7.21E-08
	PC1	0.033	0.64
	PC2	0.074	0.28

	PC3	-0.0075	0.92
	PC4	-0.05	0.47
**UKBB**	(Intercept)	-0.026	0.072
	Age	0.3	0.029
	Age^2	-0.35	0.011
	Gender	0.055	0.017
	ICV	0.63	<1E-176
	PC1	0.022	0.022
	PC10	-0.0035	0.71
	PC2	0.013	0.18
	PC3	0.00013	0.99
	PC4	-0.019	0.047
	PC5	0.004	0.67
	PC6	0.012	0.22
	PC7	-0.011	0.23
	PC8	0.0093	0.32
	PC9	-0.012	0.19
**VETS**	(Intercept)	-0.024	0.64
	Age	0.027	0.7
	Age^2	0.11	0.1
	ICV	0.58	9.88E-26
	PC1	-0.03	0.58
	PC2	-0.018	0.74
	PC3	-0.069	0.2
	PC4	-0.012	0.83

F) Putamen

Cohort	Covariate	Beta	P-Value
**MIRE**	(Intercept)	-0.26	0.22
	Age	-0.42	0.42
	Age^2	0.039	0.94
	Gender	0.29	0.2
	ICV	0.42	8.10E-09
	PC1	-0.042	0.54
	PC2	0.045	0.49
	PC3	0.14	0.033
	PC4	0.042	0.53

**PGC-PTSD**	(Intercept)	1.01	4.14E-07
	Age	-0.26	0.38
	Age^2	-0.11	0.7
	Gender	0.37	0.0046
	ICV	0.65	1.27E-23
	PC1	0.042	0.29
	PC2	-0.03	0.46
	PC3	0.027	0.48
	PC4	0.092	0.02
	siteMinneapolis	-2.11	8.69E-34
	siteNSW	-0.73	1.04E-05
	siteU Wash	-2.24	1.49E-12
**TRAC**	(Intercept)	-0.27	0.36
	Age	-0.18	0.72
	Age^2	-0.18	0.71
	Gender	0.29	0.34
	ICV	0.34	1.56E-05
	PC1	0.1	0.15
	PC2	0.069	0.3
	PC3	0.037	0.59
	PC4	-0.003	0.96
**UKBB**	(Intercept)	-0.13	1.27E-18
	Age	0.042	0.77
	Age^2	-0.27	0.052
	Gender	0.28	2.86E-31
	ICV	0.48	<1E-176
	PC1	0.01	0.3
	PC10	-0.00074	0.94
	PC2	0.0053	0.59
	PC3	-0.0085	0.38
	PC4	-0.012	0.21
	PC5	-6.30E-05	0.99
	PC6	-0.00084	0.93
	PC7	0.0063	0.52
	PC8	0.011	0.24
	PC9	-0.017	0.074
**VETS**	(Intercept)	0.0048	0.94

	Age	-0.013	0.88
	Age^2	0.048	0.58
	ICV	0.37	1.83E-10
	PC1	0.034	0.64
	PC2	-0.031	0.66
	PC3	0.044	0.52
	PC4	-0.055	0.43

G) Thalamus

Cohort	Covariate	Beta	P-Value
**MIRE**	(Intercept)	0.036	0.86
	Age	-0.12	0.81
	Age^2	-0.14	0.78
	Gender	-0.04	0.85
	ICV	0.59	1.91E-15
	PC1	-0.06	0.35
	PC2	-0.072	0.25
	PC3	0.024	0.7
	PC4	0.17	0.0065
**PGC-PTSD**	(Intercept)	0.091	0.54
	Age	0.32	0.16
	Age^2	-0.46	0.034
	Gender	0.11	0.27
	ICV	0.58	8.65E-31
	PC1	-0.0054	0.86
	PC2	0.035	0.26
	PC3	-0.025	0.4
	PC4	0.015	0.6
	siteMinneapolis	0.14	0.22
	siteNSW	-0.72	1.47E-08
	siteU Wash	-0.5	0.032
**TRAC**	(Intercept)	-0.069	0.79
	Age	0.23	0.61
	Age^2	-0.35	0.43
	Gender	0.074	0.79
	ICV	0.6	2.73E-15
	PC1	-0.026	0.67

	PC2	0.13	0.036
	PC3	-0.0065	0.92
	PC4	0.021	0.73
**UKBB**	(Intercept)	-0.019	0.13
	Age	-0.0071	0.95
	Age^2	-0.31	0.0092
	Gender	0.04	0.051
	ICV	0.65	<1E-176
	PC1	0.031	0.00016
	PC10	-0.0034	0.68
	PC2	-0.025	0.0023
	PC3	0.00098	0.91
	PC4	-0.0064	0.44
	PC5	0.012	0.15
	PC6	-0.0074	0.37
	PC7	7.20E-05	0.99
	PC8	0.013	0.12
	PC9	-0.0016	0.84
**VETS**	(Intercept)	-0.05	0.36
	Age	-0.14	0.056
	Age^2	-0.0082	0.91
	ICV	0.55	2.06E-23
	PC1	-0.088	0.15
	PC2	0.025	0.68
	PC3	-0.08	0.18
	PC4	-0.054	0.35

Abbreviations: PGC-PTSD=Psychiatric Genetics Consortium-Posttraumatic Stress Disorder, TRAC= Translational Research Center for TBI and Stress, VETS=Vietnam Era Twin Study of Aging; MIRE= MIRE=Duke University and VA Mid-Atlantic Mental Illness Research Education and Clinical Center, UKBB=United Kingdom BioBank.

Supplementary Table 3: Meta-analysis association between polygenic scores based on the Hibar et al. 2015 subcortical volume GWAS and corresponding subcortical volume (see Table 2 for hippocampus).

Region	Threshold	Beta	P-value
Accumbens	0.4131	0.051	1.99E-07
Amygdala	0.7431	0.040	0.13
Caudate	0.0371	0.083	1.97E-18
Pallidum	0.0001	0.024	0.0069
Putumen	0.0001	0.064	1.70E-12
Thalamus	0.0431	0.048	7.46E-10

Supplementary Table 4: Meta-analysis of the association of all subcortical volume PGSs with PTSD.

Region	Threshold	Beta	P-value
Accumbens	0.4131	0.015	0.79
Amygdala	0.7431	**0.14**	**0.011**
Caudate	0.0371	0.018	0.78
Hippocampus	0.8891	0.10	0.081
Pallidum	0.0001	-0.023	0.74
Putamen	0.0001	0.037	0.50
Thalamus	0.0431	0.11	0.12

Supplementary Table 5. Significance of GxE interactions between PGSs for subcortical volumes and PTSD and Childhood Trauma.

Region & Score	Threshold	PS x PTSD Beta	PS x PTSD P-value	PS x CT Beta	PS x CT P-value
Accumbens	0.4131	0.061	0.43	-0.029	0.56
Amygdala	0.7431	0.047	0.28	-0.004	0.85
Caudate	0.0371	0.073	0.10	0.077	0.41
Hippocampus	0.8891	**-0.10**	**0.027**	-0.039	0.10
Pallidum	0.0001	-0.011	0.83	-0.040	0.086
Putamen	0.0001	0.017	0.72	0.020	0.38
Thalamus	0.0431	-0.012	0.78	-0.018	0.36

Supplementary Table 6. Top GxE GWAS associations for A) the hippocampus and amygdala, and B) other subcortical regions. Only the most significant variant per gene/region is reported.

A	Region	E	Genes	Marker	CHR	POS	Effect Allele	Alt. Allele	Beta	P-Value	Direction*
	Amygdala	CT		rs4702973	5	99,293,221	a	c	17.11	2.16E-07	+++++
	Hippocampus	CT		rs75859483	8	142,956,633	a	g	-44.87	4.49E-07	----?
	Hippocampus	PTSD		rs35700850	3	8,116,965	t	tag	-32.53	9.63E-07	---+?
	Hippocampus	PTSD		rs7235463	18	48,988,864	t	c	-30.34	1.18E-06	-----
	Amygdala	PTSD	SNTG1	rs147964805	8	51,219,220	a	at	-35.29	1.19E-06	--?-?
	Hippocampus	CT	AC007204.1; ZNF253; ZNF93	rs1469702	19	19,970,787	t	g	36.38	1.43E-06	++?+?
	Amygdala	CT		rs10443966	10	26,637,408	a	g	-25.76	1.61E-06	--+-?
	Hippocampus	PTSD	KIF7; PLIN1; TICRR	rs893725	15	90,128,223	a	c	-29.45	1.64E-06	+--+-
	Amygdala	PTSD	CHST11	rs10861272	12	105,110,538	a	g	27.27	1.78E-06	?++?+
	Hippocampus	PTSD	ANO4	rs7974164	12	101,358,282	t	c	-36.82	1.83E-06	+----

B	Region	E	Genes	Marker	CHR	POS	Effect Allele	Alt. Allele	Beta	P-Value	Direction*
	Accumbens	PTSD		rs2348408	4	160,398,945	a	c	-7.54	1.47E-07	---+-
	Accumbens	CT	RP11-1085N6.3	rs17091959	14	57,157,766	t	c	-8.61	1.56E-07	--+-?
	Caudate	CT	CSMD2	rs71647939	1	34,040,737	a	g	-46.65	4.17E-07	----?
	Thalamus	CT	ALG14; RWDD3; TMEM56; TMEM56-RWDD3	rs12755552	1	95,685,026	a	g	-83.08	6.95E-07	--+--
	Caudate	PTSD	RGS13	rs10489877	1	192,570,150	a	g	-40.20	7.23E-07	--++-
	Caudate	PTSD	DHFR; MSH3; MTRNR2L2	rs3776967	5	80,002,350	t	c	-33.66	9.04E-07	-----
	Pallidum	PTSD	BDP1; MCCC2	5:70880199:G:T	5	70,880,199	t	g	-44.13	9.60E-07	-?--?
	Caudate	CT		rs72430984	2	147,063,647	a	atg	-34.86	1.12E-06	----?
	Pallidum	CT	LARS; PLAC8L1; POU4F3; RBM27; SH3RF2	rs68033861	5	14,567,1805	ct	c	-17.60	1.67E-06	+-?-?
	Pallidum	CT	RBFOX1	rs4375678	16	7,262,965	t	c	-16.40	1.68E-06	----?

*Direction: Direction of association for PCG-PTSD, UKBB, TRAC, VETS, MIRE respectively.

Supplementary Table 7. Nominally significant GxE candidate variant associations for A) the hippocampus and amygdala, and B) other subcortical regions.

A

Paper	E	Region	Gene	Marker	CHR	POS	Effect Allele	Alt. Allele	Beta	P-Value	Direction*
Nievergelt et al.	CT	Hippocampus	KAZN; TMEM51- AS1	rs148757321	1	15,436,223	ctgtg	c	24.89	0.0030	++++?
Stein et al.	CT	Hippocampus	MAD1L1	rs55789728	7	2,107,649	a	g	-17.50	0.019	----?
Stein et al.	PTSD	Hippocampus	CAMKV	rs2777888	3	49,898,000	a	g	-14.45	0.025	?--?+
Stein et al.	CT	Amygdala	CAMKV	rs2777888	3	49,898,000	a	g	7.07	0.029	?+-?-
Stein et al.	PTSD	Hippocampus	TSNARE1	rs13262595	8	143,316,970	a	g	-12.75	0.042	+-+--
Stein et al.	PTSD	Hippocampus	TSNARE1	rs4129585	8	143,312,933	a	c	-12.66	0.044	+-+--

B

Paper	E	Region	Gene	Marker	CHR	POS	Effect Allele	Alt. Allele	Beta	P-Value	Direction*
Satizabal et al.	PTSD	Pallidum	RP11-624C23.1	rs196807	8	24,682,649	a	g	12.46	0.0035	++++-
Stein et al.	PTSD	Caudate	CRHR1	rs242925	17	43,888,866	t	c	-20.04	0.0050	--+-?
Stein et al.	PTSD	Putamen	PIK3CG	rs11773880	7	106,540,171	t	g	-25.19	0.0082	---+-
Stein et al.	PTSD	Accumbens	TSNARE1	rs13262595	8	143,316,970	a	g	-3.37	0.013	----+
Stein et al.	PTSD	Accumbens	PIK3CG	rs11773880	7	106,540,171	t	g	-3.85	0.013	+-++-
Stein et al.	PTSD	Accumbens	TSNARE1	rs4129585	8	143,312,933	a	c	-3.35	0.014	----+
Stein et al.	PTSD	Accumbens	TCF4	rs35371867	18	53,193,027	a	g	-3.33	0.018	--+-?
Stein et al.	PTSD	Accumbens	CRHR1	rs242925	17	43,888,866	t	c	-3.21	0.021	--+-?
Stein et al.	PTSD	Caudate	MAD1L1	rs7680	7	1,855,531	a	g	-21.39	0.023	---+?
Nievergelt et al.	PTSD	Accumbens	PARK2	rs9364611	6	162,163,506	t	c	4.86	0.025	++-+?
Stein et al.	CT	Thalamus	MAD1L1	rs55789728	7	2,107,649	a	g	-24.66	0.026	----?
Stein et al.	PTSD	Lat. Ventricle	MAD1L1	rs10235664	7	2,086,814	t	c	243.77	0.029	+++-+
Stein et al.	PTSD	Lat. Ventricle	MAD1L1	rs55789728	7	2,107,649	a	g	262.99	0.030	+++-?
Satizabal et al.	CT	Putamen	DLG2; LOC107984425	rs1432054	11	83,260,225	a	g	17.63	0.040	++-+?
Stein et al.	CT	Accumbens	CAMKV	rs2777888	3	49,898,000	a	g	2.80	0.041	?++?-

*Direction: Direction of association for PCG-PTSD, UKBB, TRAC, VETS, MIRE respectively.
